# The Diagnostic Puzzle of Guillain-Barré Syndrome Following a Viral Prodrome: A Case Report Highlighting the Role of CSF and Electrophysiology

**DOI:** 10.7759/cureus.94252

**Published:** 2025-10-09

**Authors:** Nandar Eaindray Khin, Theint Shwe Yi Win, Lin Lin Tun Myat

**Affiliations:** 1 Acute Medicine, Sherwood Forest NHS Trust, Sutton In Ashfield, GBR; 2 Orthogeriatrics, Leeds Teaching Hospital NHS Trust, Leeds, GBR; 3 Health Education Thames Valley (HETV), Royal College of Medicine, London, GBR

**Keywords:** acute inflammatory demyelinating polyneuropathy, guillain-barré syndrome, immunoglobulin therapy, nerve conduction studies, physiotherapy

## Abstract

Guillain-Barré Syndrome (GBS) is an acute immune-mediated polyneuropathy often triggered by a viral or gastrointestinal infection. Prompt recognition and treatment are vital to prevent morbidity and complications such as respiratory failure. We present a diagnostically challenging case of a 69-year-old male with hypertension, diabetes, and atrial fibrillation who developed progressive limb weakness and recurrent falls following a self-limiting flu-like illness. Initial clinical, laboratory, and imaging evaluations were non-specific. The lack of cranial nerve involvement and the absence of clear sensory deficits contributed to diagnostic uncertainty. Cerebrospinal fluid (CSF) analysis revealed albuminocytologic dissociation, and nerve conduction studies confirmed acute inflammatory demyelinating polyneuropathy (AIDP). Early initiation of intravenous immunoglobulin (IVIG), prior to electrophysiological confirmation, led to marked clinical improvement. This case highlights the importance of considering GBS despite atypical features, the diagnostic value of CSF analysis, and the benefit of early IVIG therapy.

## Introduction

Guillain-Barré Syndrome (GBS) is a rare but serious autoimmune disorder characterized by acute flaccid paralysis, typically involving symmetrical weakness, diminished or absent reflexes, and variable sensory symptoms. The most common variant in Western countries is acute inflammatory demyelinating polyneuropathy (AIDP) [1,2]. The global incidence of GBS is estimated at 1-2 cases per 100,000 person-years, with a slight male predominance and increasing risk with age [1]. It is classically preceded by a respiratory or gastrointestinal illness, after which patients develop subacute neurological symptoms over days to weeks.

Diagnosis is primarily clinical, supported by cerebrospinal fluid (CSF) findings of albuminocytologic dissociation and nerve conduction studies (NCS) indicating demyelination. However, atypical presentations, preserved sensation, and unremarkable neuroimaging can complicate diagnosis. Early treatment with intravenous immunoglobulin (IVIG) or plasmapheresis significantly improves outcomes, particularly when initiated within two weeks of symptom onset [3,4]. Several studies also support initiating IVIG based on clinical suspicion alone, without awaiting electrophysiological confirmation [5-7].

This case is noteworthy because the patient presented with preserved sensation, normal imaging, and non-specific laboratory findings--features that obscured early diagnosis. Given that atypical or motor-predominant forms may lead to delayed recognition and treatment, this report aims to emphasize the clinical reasoning, use of supportive investigations, and timely initiation of IVIG that contributed to diagnostic clarity and recovery, underscoring the importance of maintaining clinical suspicion even in non-classical GBS presentations.

## Case presentation

A 69-year-old male with a past medical history of hypertension, type 2 diabetes mellitus, and atrial fibrillation presented to the emergency department after experiencing five days of progressive lower limb weakness, resulting in recurrent falls. He also reported lower back, buttock, and thigh pain. Two weeks earlier, he experienced flu-like symptoms, including low-grade fever, myalgia, and malaise, which resolved spontaneously. He denied chest pain, breathlessness, dysphagia, urinary or fecal incontinence, and had no recent travel history.

On examination, his vital signs were within normal limits. Neurological evaluation revealed the following.

Muscle strength (Medical Research Council (MRC) scale, 0-5): Upper limbs - shoulder abduction 3/5, elbow flexion 3/5, wrist extension 3/5; Lower limbs - hip flexion 2/5, knee extension 2/5, ankle dorsiflexion 2/5. MRC sum score: 22/60. Reflexes: Absent (0) at biceps, triceps, patellar, and Achilles tendons bilaterally. Sensation: Intact to light touch, pinprick, vibration, and proprioception.Cranial nerves and cerebellar function: No abnormalities detected.

The patient was unable to stand or ambulate independently.

Diagnostic evaluation

Laboratory investigations were largely unremarkable, with normal full blood count, renal and liver function, and inflammatory markers (C-reactive protein (CRP), erythrocyte sedimentation rate (ESR)). The autoimmune panel, including antineutrophil cytoplasmic antibodies (ANCA) and anticardiolipin antibodies, was within normal limits (Table 1). Creatine kinase was mildly elevated (272 U/L). COVID-19 testing was not performed.

**Table 1:** Antineutrophil cytoplasmic antibody (ANCA) panel and cardiolipin antibody screen

Test	Patient Value	Normal Range	Interpretation
Anti-MPO	14.0 CU	0 – 19.9 CU	Within normal range
Anti-PR3	2.4 CU	0 – 19.9 CU	Within normal range
Cardiolipin antibody (IgG/IgM/IgA)	Negative	Negative	Normal

On Day 5 of hospital admission, a lumbar puncture revealed markedly elevated CSF protein (2367 mg/L) with a low white cell count (4 cells/μL) and normal glucose, consistent with albuminocytologic dissociation. Infectious screening of the CSF was negative. In addition, oligoclonal bands were present but demonstrated identical patterns in serum and CSF, indicating a systemic inflammatory process rather than intrathecal synthesis (Table 2). The constellation of progressive symmetrical weakness, areflexia, and these CSF findings supported a provisional diagnosis of GBS, even while electrophysiological studies were still pending. Further antibody testing, including cerebellar antibodies (Table 3), glycolipid antibodies (IgM and IgG; Table 4), and neuronal and Purkinje cell antibodies (Table 5), was negative.

**Table 2:** Cerebrospinal fluid (CSF) analysis Elevated CSF protein. Oligoclonal bands are identical in serum and CSF, suggesting a systemic inflammatory process.

Parameter	Patient Value	Normal Range	Interpretation
Appearance	Clear and colorless	Clear and colorless	Normal
Red blood cells	15 /µL	<5 /µL	Elevated
White blood cells	4 /µL	<5 /µL	Normal
Organisms	None seen	None	Normal
Culture	No growth after 48 hrs	No growth	Normal
CSF Protein	2367 mg/L	150–450 mg/L	High
Oligoclonal Bands (HRE)	Identical patterns in serum & CSF	Normally absent	Present

**Table 3:** Cerebellar antibodies

Test	Patient Result	Normal Range	Interpretation
Anti-Hu	Negative	Negative	Normal
Anti-Yo	Negative	Negative	Normal
Anti-Ri	Negative	Negative	Normal
Anti-Amphiphysin	Negative	Negative	Normal

**Table 4:** Glycolipid antibody (IgM & IgG) findings

Test	Patient Result	Reference Range
GM1 IgM	<500	<500
GD1a IgM	<500	<500
GD1b IgM	<500	<500
GQ1b IgM	<500	<500
GM2 IgM	<500	<500
GM1 IgG	<500	<500
GM2 IgG	<500	<500
GD1a IgG	<500	<500
GD1b IgG	<500	<500
GQ1b IgG	<500	<500

**Table 5:** Neuronal antibody assay No evidence of anti-neuronal or anti-Purkinje cell staining.

Test	Patient Result	Reference Range
Anti-neuronal antibody	Negative	Negative
Anti-Purkinje cell antibody	Negative	Negative

The presence of oligoclonal bands with identical patterns in both serum and CSF indicates systemic immune activation rather than intrathecal synthesis. This distinction is clinically important, as it helps exclude primary CNS demyelinating conditions, such as multiple sclerosis, and reinforces the peripheral nature of the pathology in this case.

**Figure 1:**
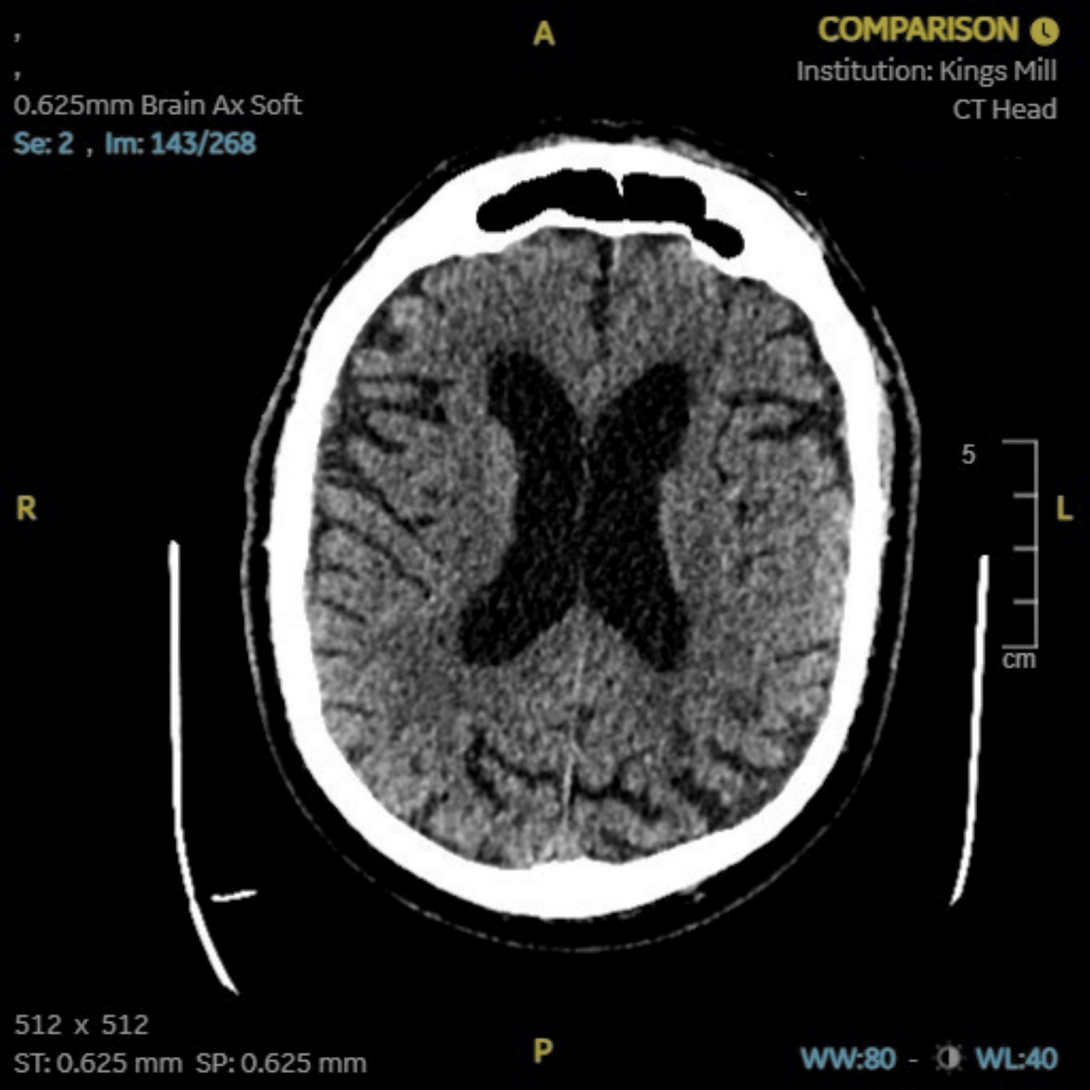
CT scan of the head showing no acute intracranial pathology

**Figure 2:**
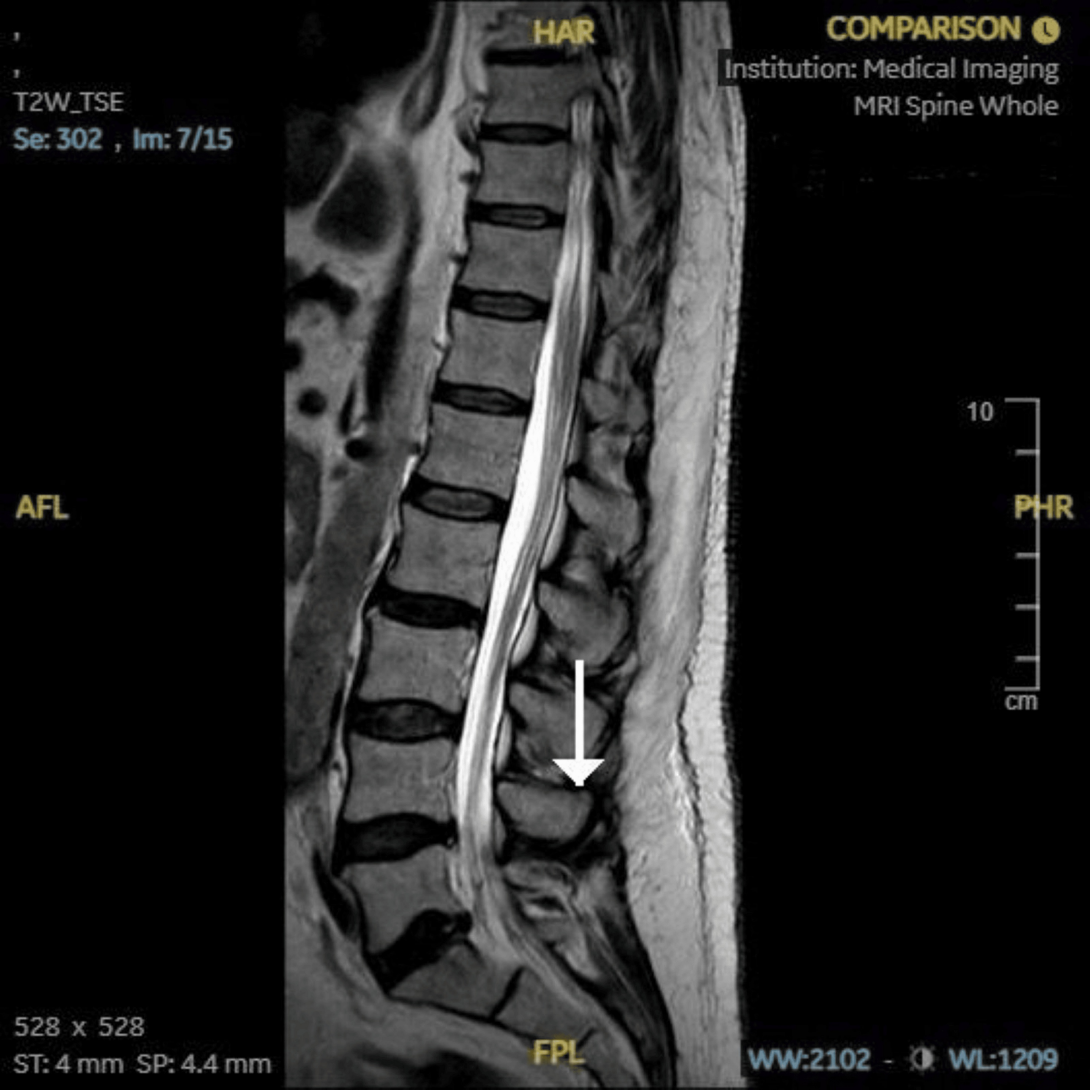
MRI of the lumbar spine (sagittal T2-weighted sequence) demonstrating preserved vertebral alignment and no abnormal signal changes. A white arrow highlights the lumbar spine region of interest.

**Figure 3:**
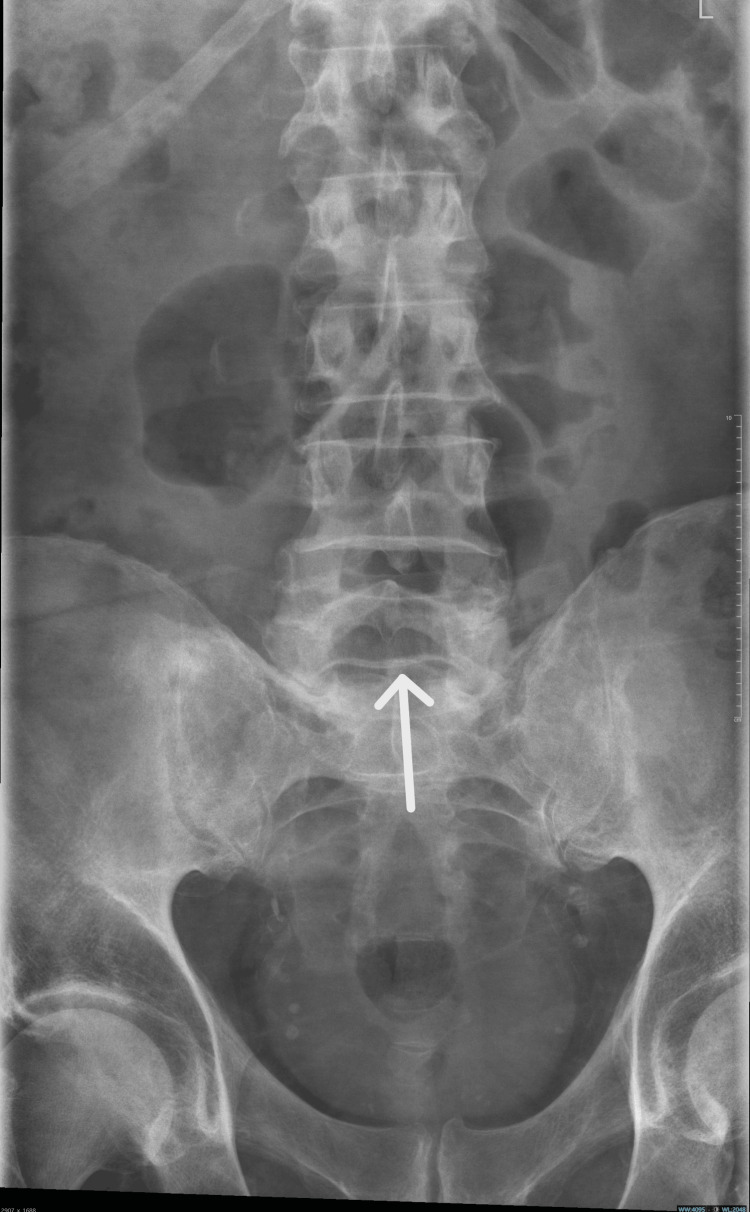
Anteroposterior lumbar spine X-ray showing minor degenerative changes at the lower lumbar levels (arrow), with preserved vertebral alignment

Neuroimaging studies were obtained to exclude alternative diagnoses that could mimic the patient’s presentation. A CT scan of the head (Figure 1) showed no acute intracranial pathology, ruling out stroke or structural brain lesions. MRI of the spine (Figure 2) revealed no evidence of myelopathy or compressive lesions. A lumbar spine X-ray (Figure 3) demonstrated only minor degenerative changes, without findings to explain the acute neurological deficit. These results supported a peripheral rather than central etiology for the patient’s weakness.

On Day 11, NCS confirmed acute inflammatory demyelinating polyneuropathy (AIDP). Sensory conduction studies showed absent sural responses and reduced amplitudes in the superficial peroneal nerve (Table 6). Motor conduction studies demonstrated reduced amplitudes and velocities, with conduction block in the left common peroneal nerve and temporal dispersion in the left tibial nerve (Table 7). F-wave studies revealed prolonged latencies in the tibial nerve, consistent with demyelination (Table 8). Repetitive stimulation did not demonstrate significant decrement or facilitation (Table 9).

**Table 6:** Sensory NCS results of the patient Distances are in centimeters; latencies in milliseconds; amplitudes in microvolts; conduction velocities in meters/second. R = right; L = left; Sup = superficial; Lat = lateral; NR = not recordable; NCS = nerve conduction study

Nerve / Sites	Recording Site	Latency (ms)	Peak Amplitude (µV)	Distance (cm)	Velocity (m/s)
R Median – Ulnar (Digit II)	Wrist	2.76	7.5	13.5	48.9
R Median – Ulnar (Digit V)	Wrist	2.24	3	11	49.1
R Sural – Lat Malleolus	Calf	NR	NR	—	—
L Sural – Lat Malleolus	Calf	NR	NR	—	—
R Sup Peroneal – Foot	Lateral Leg	3.13	3.5	12.5	40

**Table 7:** Motor NCS Motor NCS results demonstrating reduced conduction velocity and amplitude in multiple nerves. Conduction block observed in the left common peroneal nerve; temporal dispersion noted in the left tibial nerve. Latencies are in milliseconds; amplitudes in millivolts. R = right; L = left; APB = abductor pollicis brevis; ADM = abductor digiti minimi; EDB = extensor digitorum brevis; Tib Ant = tibialis anterior; AH = abductor hallucis; Fib Head = fibular head; NCS = nerve conduction study

Nerve / Sites	Recording Site	Latency (ms)	Amplitude (mV)	Distance (cm)	Velocity (m/s)
R Median – APB	Wrist	4.53	8.9	—	—
	Elbow	10.36	7.2	29	49.7
R Ulnar – ADM	Wrist	3.28	7.8	—	—
	Below Elbow	8.44	8.4	23	44.6
R Common Peroneal – EDB	Ankle	5.47	0.6	—	—
L Common Peroneal – EDB	Ankle	5.26	1.9	—	—
	Fib Head	18.75	0.3	35	25.9
R Common Peroneal – Tib Ant	Fib Head	3.7	1.3	—	—
	Knee	7.29	1.1	10	27.8
L Common Peroneal – Tib Ant	Fib Head	4.22	1.7	—	—
	Knee	6.82	1.5	9	34.6
R Tibial Malleolus – AH	Ankle	4.9	1.9	—	—
L Tibial Malleolus – AH	Ankle	4.48	1.1	—	—

**Table 8:** F-wave study Prolonged F-wave latencies were noted in the tibial nerve, consistent with demyelination. R = right; AH = abductor hallucis; APB = abductor pollicis brevis; ADM = abductor digiti minimi; Lat = lateral

Nerve	Min F Lat (ms)	Max F Lat (ms)	Mean F Lat (ms)
R Tibial Malleolus – AH	60.52	91.82	71.24
R Median – APB	26.82	26.98	26.91
R Ulnar – ADM	39.58	41.09	40.51

**Table 9:** Repetitive nerve stimulation (RepStim) Repetitive stimulation study did not demonstrate significant decrement or facilitation. Δ = change; Fac = facilitation; Area measured in mV·ms; R = right; Abd Poll Brevis = abductor pollicis brevis; Abd Dig Min = abductor digiti minimi; pps = pulses per second

Muscle / Train	Amplitude (mV)	Δ Amp1 (%)	Δ Amp2 (%)	Fac Amp (%)	Area (mVms)	Δ Area1 (%)	Δ Area2 (%)	Fac Area (%)	Rate (pps)	Time
R Abd Poll Brevis (Rest)	9.3	–0.9	–0.6	100	32	–2.6	–3.4	100	3	0:00:00
R Abd Dig Min (Baseline)	9.1	–0.5	–2.1	100	38.3	–7.4	–7.6	100	3	0:00:00

Management and outcome

Treatment with a five-day course of IVIG (0.4 g/kg/day) was initiated on Day 5 immediately following the CSF results. Physiotherapy began concurrently. Respiratory function and swallowing ability were monitored regularly; no decline was noted. By Day 13, the patient demonstrated significant improvement in limb strength and mobility. At discharge on Day 17, he was able to walk short distances with assistance, with an MRC sum score of 54/60, and was discharged with a structured outpatient rehabilitation plan. The overall clinical timeline, including initial presentation, diagnostic workup, treatment, and recovery milestones, is summarized in Table 10.

**Table 10:** Clinical timeline GBS: Guillain-Barré Syndrome; IVIG: intravenous immunoglobulin; NCS: nerve conduction studies; AIDP: acute inflammatory demyelinating polyneuropathy; PT: physiotherapy

Day	Event	Findings	Impression
-14	Viral prodrome	Flu-like illness	Presumed upper respiratory infection
-5	Symptom onset	Progressive limb weakness	Possible spinal or neuromuscular etiology
0	ED presentation	LL 2/5, UL 3/5, areflexia, sensation intact	?GBS, ?myopathy
1	Imaging	Normal CT head and MRI spine	Structural causes excluded
5	Lumbar puncture	Elevated protein, low WBC	Suspicious for GBS
5	CSF organism	Negative	Infectious etiology unlikely
5	IVIG initiated	5-day course + PT	Suspicious clinical diagnosis of GBS
11	NCS	Demyelinating neuropathy	AIDP confirmed
13	Clinical improvement	Improved strength	Favorable response to therapy
17	Discharge	With a rehabilitation plan	Recovery underway

## Discussion

GBS remains a diagnostic challenge because of its heterogeneous presentations and overlap with other neuromuscular conditions. Differential diagnoses, such as spinal cord compression, inflammatory myopathies, and myasthenia gravis, were excluded based on imaging and laboratory evaluation. In our case, the diagnostic process was complicated by a motor-predominant presentation without sensory involvement, normal neuroimaging, and incidental laboratory findings, which could have delayed recognition. Such atypical features underscore the importance of maintaining a high index of suspicion, particularly following a recent viral prodrome.

GBS encompasses several variants, including the acute motor axonal neuropathy (AMAN) and acute motor-sensory axonal neuropathy (AMSAN) subtypes, which may present with motor-predominant or sensory-sparing features similar to our case. These can mimic other neuromuscular disorders, such as myasthenia gravis, spinal cord compression, or inflammatory myopathies; however, the combination of areflexia, CSF albuminocytologic dissociation, and demyelinating features on NCS supported the diagnosis of AIDP in this patient.

In this patient, CSF findings of albuminocytologic dissociation, combined with clinical assessment, enabled the early initiation of IVIG without awaiting confirmatory NCS results.

In GBS, albuminocytologic dissociation is a hallmark finding, occurring in up to 90% of cases by the second week [2]. Although electrophysiological studies provide confirmatory evidence for GBS, they may lag behind clinical presentation. Therefore, a normal nerve conduction study early in the disease course does not exclude the diagnosis, and clinical suspicion, supported by CSF analysis, should guide timely management [3]. Reliance on NCS before initiating treatment may unnecessarily delay effective therapy.

Objective measures documented the patient’s recovery: the MRC sum score increased from 22/60 to 54/60, demonstrating substantial functional improvement. Comparison with previously reported motor-predominant or atypical GBS cases highlights both similarities, such as reliance on electrophysiology and CSF for diagnosis, and distinctive features of our case, reinforcing its educational and scientific value.

Current guidelines recommend initiating IVIG or plasmapheresis within two weeks of symptom onset [4,8]. Several studies reinforce this approach. Coll-Cantí et al. demonstrated that patients treated with IVIG within five days had significantly shorter hospital stays compared to those treated later or not at all [5]. A Korean multicenter cohort study also showed that early IVIG initiation correlated with better functional outcomes and faster recovery [6]. Similarly, Cochrane reviews and expert consensus emphasize that early treatment improves prognosis, reduces complications, and shortens disease duration [7]. More recently, the European Academy of Neurology/Peripheral Nerve Society (EAN/PNS) 2023 guideline similarly recommends early initiation of IVIG or plasma exchange in patients unable to walk independently within two weeks of weakness onset and emphasizes the role of CSF and electrodiagnostic studies in atypical or motor-predominant cases [8].

This patient's favorable response to IVIG further supports early immunotherapy based on clinical suspicion and CSF findings. A multidisciplinary approach involving neurologists, internal medicine physicians, infectious disease specialists, and rehabilitation teams is also essential to ensure comprehensive management, monitor for complications, and facilitate functional recovery in atypical presentations.

## Conclusions

GBS should be suspected in patients presenting with progressive symmetrical weakness following recent infections, particularly when sensory function and neuroimaging are normal, as these features can obscure early recognition. CSF analysis and timely clinical assessment are critical for diagnosis. Initiating IVIG based on clinical suspicion and CSF findings--without awaiting nerve conduction confirmation--can significantly improve outcomes. This case highlights the importance of early intervention and clinical vigilance in atypical, motor-predominant GBS presentations.
